# Unveiling high specific energy supercapacitor from layer-by-layer assembled polypyrrole/graphene oxide|polypyrrole/manganese oxide electrode material

**DOI:** 10.1038/s41598-019-41203-3

**Published:** 2019-03-20

**Authors:** Shalini Kulandaivalu, Nadhrah Suhaimi, Yusran Sulaiman

**Affiliations:** 1 Department of Chemistry, Faculty of Science, Universiti Putra Malaysia, 43400 Serdang, Selangor Malaysia; 2 Functional Devices Laboratory, Institute of Advanced Technology, Universiti Putra Malaysia, 43400 Serdang, Selangor Malaysia

## Abstract

A novel layer-by-layer (LBL) based electrode material for supercapacitor consists of polypyrrole/graphene oxide and polypyrrole/manganese oxide (PPy/GO|PPy/MnO_2_) has prepared by electrochemical deposition. The formation of LBL assembled nanocomposite is confirmed by Fourier transform infrared spectroscopy, Raman spectroscopy and X-ray diffraction. The field emission scanning electron microscopy images clearly showed that PPy/MnO_2_ was uniformly coated on PPy/GO. The PPy/GO|PPy/MnO_2_ symmetrical supercapacitor has revealed outstanding supercapacitive performance with a high specific capacitance of 786.6 F/g, an exceptionally high specific energy of 52.3 Wh/kg at a specific power of 1392.9 W/kg and preserve a good cycling stability over 1000 cycles. It is certain that PPy/GO|PPy/MnO_2_ has an extraordinary perspective as an electrode for future supercapacitor developments. This finding contributes to a significant impact on the evolution of electrochemical supercapacitor.

## Introduction

The search and strong demand for long-lasting energy security is mainspring for the development of energy storage systems. In particular, of all energy storage systems, electrochemical supercapacitors (SCs) set its own perspectives and expectations by bridging the gap between batteries and conventional capacitors. However, certain limitations of SC need to be overcome to reinforce and instill the available technologies. An ideal SC should have larger specific energy than batteries and larger specific power than conventional capacitors. Even though SC is moving towards that direction, more advances in the material are needed to achieve that position. Specifically, an increment in specific capacitance will endow supercapacitor with enhanced specific energy^1^. Thus, high specific energy can be achieved by making a device with high supercapacitive properties, which is usually achieved by materials that go through faradaic reactions. However, such materials usually suffer from poor cycling stabilities.

Up to now, conducting polymer, metal oxides/hydroxides and carbon-based materials have been the quintessential electrode materials for SC. The conducting polymer, polypyrrole (PPy) has received copious attention for its high capacitance, easy fabrication process, better thermal and chemical stability^2,3^. As one of the most competitive carbon materials, graphene oxide (GO) is studied extensively as an electrode material for SC due to good conductivity and high surface area^4,5^. Among the electroactive oxides, manganese oxides (MnO_2_) is the most studied due to its high theoretical specific capacitance, environmental compatibilities and good mechanical stability^6,7^.

Here, we are utilizing layer-by-layer (LBL) assembly as a promising approach to fabricate electrode material for SC. Basically, LBL is a process to build up a film with multiple layers by bringing in contact the substrate with solution alternatively^8^. There are various interactions involved in assembling LBL films such as electrostatic, hydrogen bonding, covalent bonding, hydrophobic interaction, van der Waals and so on^9,10^. In the most recent, some works have been conducted on LBL for development of electrode material for SC such as polyaniline/GO^11,12^, PPy/GO^13^ and poly(3,4-ethylenedioxythiophene)/ruthenium oxide^14^, in which the main idea is to bring close two different material in nanoscale level. Despite all these evolvements in electrode material, the energy output of SC still subtle. Yet, the bilayer film built up of PPy/GO composite and PPy/MnO_2_ composite for SC, to the best of our knowledge has never been reported.

Hence, in the current investigation, we have designed and assembled an electrode consisting of PPy/GO and PPy/MnO_2_ through an LBL approach. The assembled nanocomposite has various benefits. The most anticipated virtues of this LBL assembled are (1) providing an enormous number of active sides for diffusion of electrolytes in layered composite and (2) directly forming a binder-free supercapacitor electrode on a conductive substrate, in which, it would reduce the contact resistance between electrode material and substrate. Benefiting from the synergistic effect of LBL construction, this newly designed electrode material demonstrated superior specific energy, satisfactory cycling performance and enhanced specific capacitance.

## Experimental

### Chemicals

Manganese sulfate monohydrate (MnSO_4_.H_2_O) was purchased from Sigma-Aldrich. Ethanol (95%), acetone (99.5%) and graphene oxide (GO) were supplied by HmbG Chemicals, ChemAR and Graphenea, respectively. Milli- Q deionized water (Millipore, 18.2 MΩ.cm at 25 °C) was used in all experiments. Sodium sulfate (Na_2_SO_4_; 99%) and pyrrole (97%; stored at 2–8 °C) were supplied by Merck. Pyrrole was distilled prior to use and other chemicals were used without any further purifications. Indium tin oxide (ITO) glasses were purchased from Xin Yan Technology Ltd.

### Layer-by-layer assembly of PPy/GO with PPy/MnO_2_

**Figure 1:**
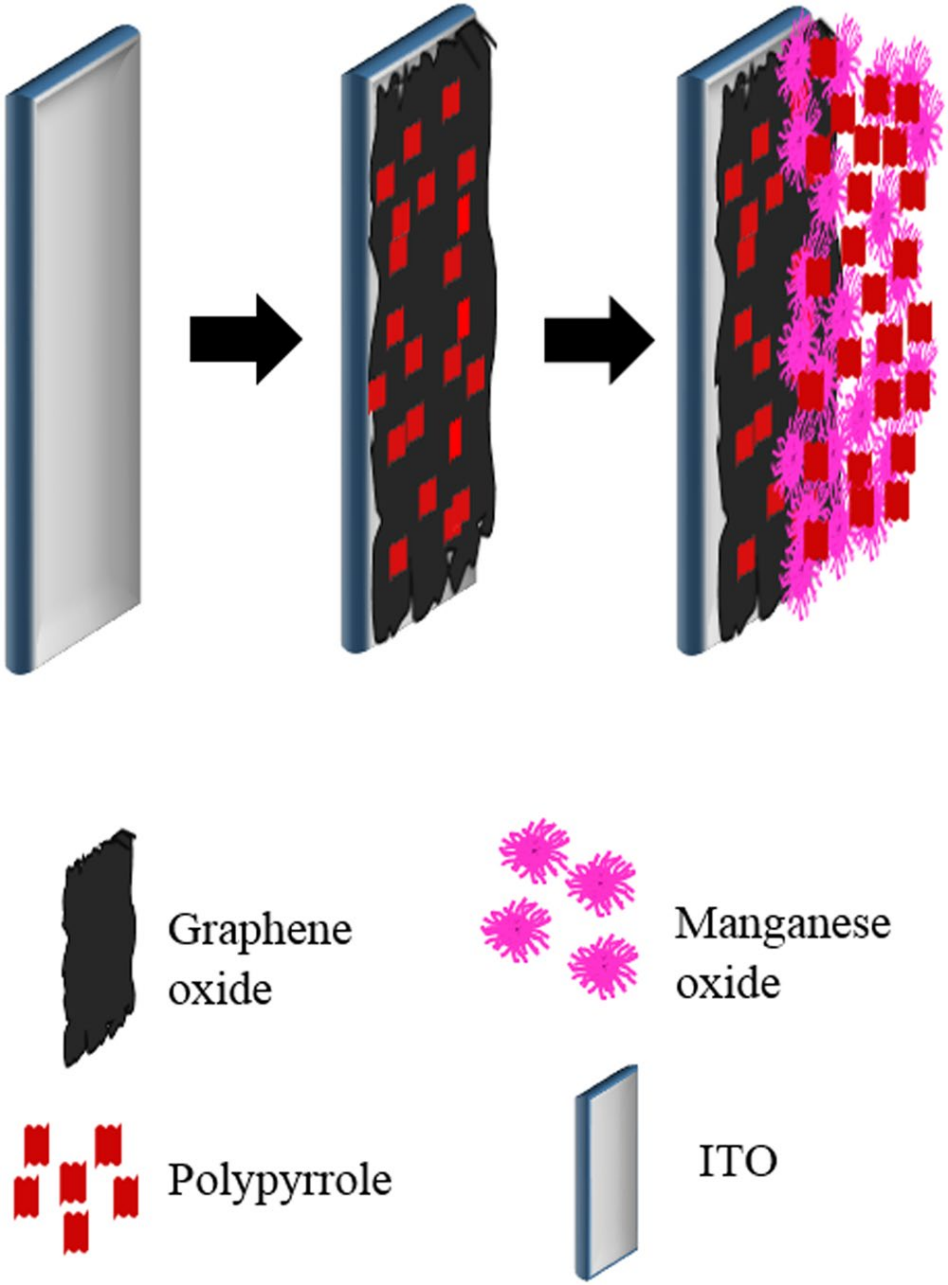
Schematic illustration of the preparation procedure of PPy/GO|PPy/MnO_2_.

LBL of PPy/GO with PPy/MnO_2_ (Fig. 1) was carried out by a simple and convenient electrochemical deposition method conducted using a Metrohm Autolab/M101 potentiostat. In brief, a three-electrode system was employed for electrodeposition, wherein ITO was used as a working electrode, platinum coil served as a counter electrode and silver/silver chloride (Ag/AgCl) worked as a reference electrode. Prior to the electrodeposition, the ITO substrate (geometrical area 1 cm^2^) was ultrasonically cleaned with ethanol, acetone and deionized water sequentially for 15 min before use. Two different solutions were prepared separately for the fabrication of LBL composite with the following procedures. 1 mg/ml GO aqueous dispersion was ultrasonicated for 60 min and was then added with 100 mM pyrrole monomer to obtain pyrrole/GO solution. Whereas, in a separate flask, 100 mM pyrrole and 0.1 M MnSO_4_.H_2_O was mixed in deionized water. For the preparation of LBL composite, first the PPy/GO film layer was electrodeposited on the cleaned ITO at a constant potential of 0.8 V for 10 minutes using chronoamperometry technique. After that, the PPy/MnO_2_ film layer was electrodeposited on the previously obtained PPy/GO layer under the same condition. For comparison, the single layer films of PPy/GO and PPy/MnO_2_ were also electrodeposited using a similar method.

### Characterization

The surface morphologies of the as-prepared nanocomposites were examined by field emission scanning electron microscopy (JEOL JSM-T600F. The presence of functional groups was detected by Fourier transform infrared (FTIR, Perkin-Elmer FT-IR spectrophotometer coupled with UATR accessory) in the frequency range from 4000 to 400 cm^−1^ and Raman spectroscopy (WITEC Alpha 300 R) in the frequency range from 4000 to 200 cm^−1^ with an excitation wavelength of 532 nm. The X-ray diffraction (XRD) patterns were recorded with a Shimadzu X-ray diffractometer with Cu Kα radiation (λ = 1.54 Å).

### Electrochemical performance

The capacitive performance was evaluated at room temperature in a two-electrode symmetrical cell assembly, which were separated by a filter paper immersed in 1.0 M Na_2_SO_4_ aqueous solution. Each sample was weighed before and after electrodeposition in order to determine the mass of deposited electroactive material on ITO. The cyclic voltammetry (CV) curves, galvanostatic charge-discharge (GCD) curves, electrochemical impedance spectroscopy (EIS) plots were obtained by using a potentiostat (Metrohm Autolab/M101). The EIS measurements were performed at open circuit potential (OCP) by using a 5 mV AC sinusoid signal in the frequency range from 100 kHz to 0.01 Hz. CV measurements were carried out at the potential window between 0 to 1 V at scan rates ranging from 25 to 200 mV/s. The GCD test was performed at different current densities (3.0 to 7.0 A/g) in the potential range of 0–1 V. The specific power, specific energy and specific capacitances were measured based on the mass of both electrodes, anode and cathode. The specific capacitance was calculated from the CV curves according to the following equation:

1 $${C}_{{\rm{sp}}}=\frac{{\int }_{{V}_{a}}^{{V}_{c}}I(V)dV}{\upsilon \times m\times ({V}_{c}-{V}_{a})}$$

where, *C*_sp_ is the specific capacitance (F/g), and *V*_a_ and *V*_c_ are the integration limits of the CV (V), *I* is the response current (A), *υ* is the potential scan rate (mV/s), and *m* is the average mass of two electrodes (g).

The specific power and specific energy were calculated from GCD curves based on the following equations:

2 $${\rm{E}}=\frac{{C}_{{\rm{sp}}}\times {\rm{\Delta }}{V}^{2}}{2}$$

3 $${\rm{P}}=\frac{{\rm{\Delta }}V\times I}{2m}$$

where, *E* is the specific energy (Wh/kg), *P* is the specific power (W/kg), *C*_sp_ is the specific capacitance (F/g), *I* is the discharge current (A), and ∆*V* is the cell operation potential (V) and *m* is the average mass of two electrodes (g).

## Results and Discussion

### Fourier Transform Infrared Spectroscopy (FTIR)

**Figure 2:**
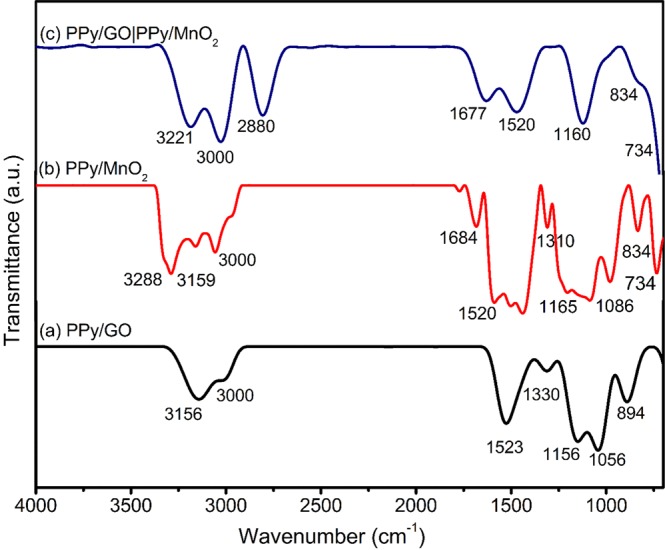
FTIR spectra of (**a**) PPy/GO, (**b**) PPy/MnO_2_ and (**c**) LBL assembled composite.

The FTIR spectra of PPy/GO, PPy/MnO_2_ and LBL assembled PPy/GO|PPy/MnO_2_, are presented in Fig. 2. In the spectrum of PPy/GO (Fig. 2a), peaks at 3000 cm^−1^ and 1056 cm^−1^ are assigned to O-H and epoxide (C–O–C) of GO, respectively^15^. While, peaks at 3156 cm^−1^ (N-H), 1523 cm^−1^ (C=C stretching), 1330 cm^−1^ (C-H plane ring deformation), 1156 cm^−1^ (C-N plane ring) and 894 cm^−1^ (polymerized pyrrole) are ascribed to the characteristic of PPy. However, the peak in the range of 1600–1700 cm^−1^ that corresponding to the C=O stretching vibration of GO is disappeared due to the formation of hydrogen bond between the carboxyl groups of GO sheets and the –NH groups of PPy^16^. As reported by Wu *et al*.^4^, the C=C stretching peak of PPy is located at 1550 cm^−1^ but this peak has shifted to 1523 cm^−1^, indicating the existence of π − π and hydrogen bonding between GO and PPy.

In the spectrum of PPy/MnO_2_ (Fig. 2b), the peak around 3000 cm^−1^ and 1684 cm^−1^ are assigned to O-H stretching and O-H bending, respectively. Whereas, the characteristic peak of MnO_2_ is noticed at 734 cm^−1^, attributed to Mn-O bond^17,18^. Despite this, all other main characteristic peaks of PPy are also seen in the spectrum which closely resembles peaks in the PPy/GO spectrum. However, a point to note here is that, after MnO_2_ is incorporated with PPy in PPy/MnO_2_, the corresponding peaks of C-N and N-H are shifted to higher frequency range compared to PPy/GO spectrum^17^.

The LBL assembled composite spectrum (Fig. 2c) shows the peaks approximately similar to the combination of vibration peaks of PPy/GO and PPy/MnO_2_ composites. Additionally, the bands of C–O–C and C=C-N deformation are disappeared which expected that have overlapped with both of peaks of PPy at around 1060 cm^−1^ and 834 cm^−1^. It is noteworthy, some of the peaks in LBL assembled composite are shifted as a result of interaction between PPy/GO and PPy/MnO_2_.

### Raman spectroscopy

**Figure 3:**
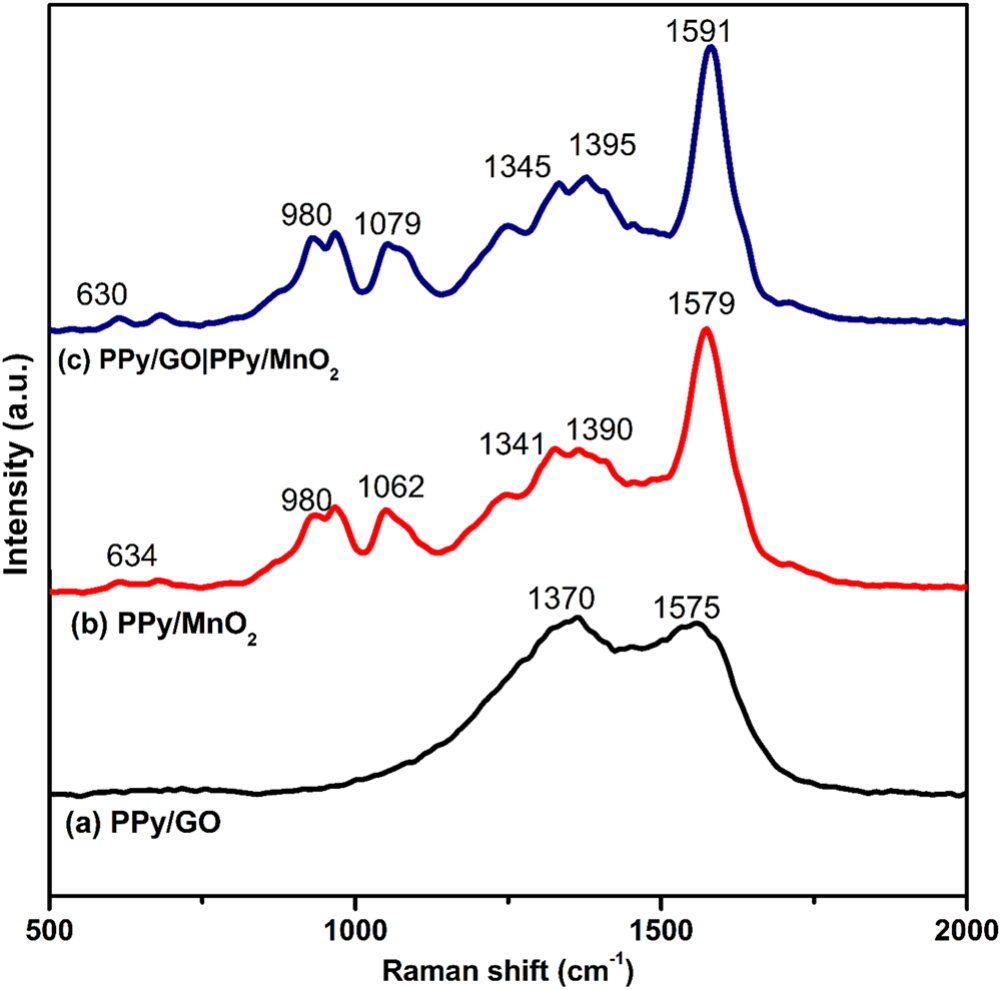
Raman spectra of (**a**) PPy/GO, (**b**) PPy/MnO_2_ and (**c**) LBL assembled composite.

The Raman analysis for the as-synthesized nanocomposites (Fig. 3) was carried out to further confirm the presence of active materials. The existence of D and G bands in PPy/GO spectrum (Fig. 3a) are observed at 1370 cm^−1^ and 1575 cm^−1^, respectively. The D band is due to vibration of aromatic rings, random edges arrangement and low symmetric carbon structure^19^. While, the G band corresponds to the first-order scattering of the E_2g_ of *sp*^2^ -bonded carbon atom^17,20^. It is observed that the vibration bands of PPy do not appear in the range of 1350 cm^−1^ to 1580 cm^−1^ due to low intensity and might be overlapped with GO peaks (G and D bands). Whereas, PPy/MnO_2_ (Fig. 3b) spectrum shows a peak at 634 cm^−1^ for the Mn–O lattice vibrations. A few broad peaks at 1579 cm^−1^ (C=C stretching), 1390 cm^−1^ (C-N stretching), 1062 cm^−1^ and 980 cm^−1^ (C-H ring deformation vibration) are associated to the characteristics of PPy^19,21^. In the spectrum of LBL assembled composite (Fig. 3c), all the characteristic peaks of PPy/MnO_2_ and PPy/GO clearly appear. However, there are some peaks shifted, which could be due to electrostatic interactions and hydrogen bonding^22^.

### X-ray diffractometry (XRD)

**Figure 4:**
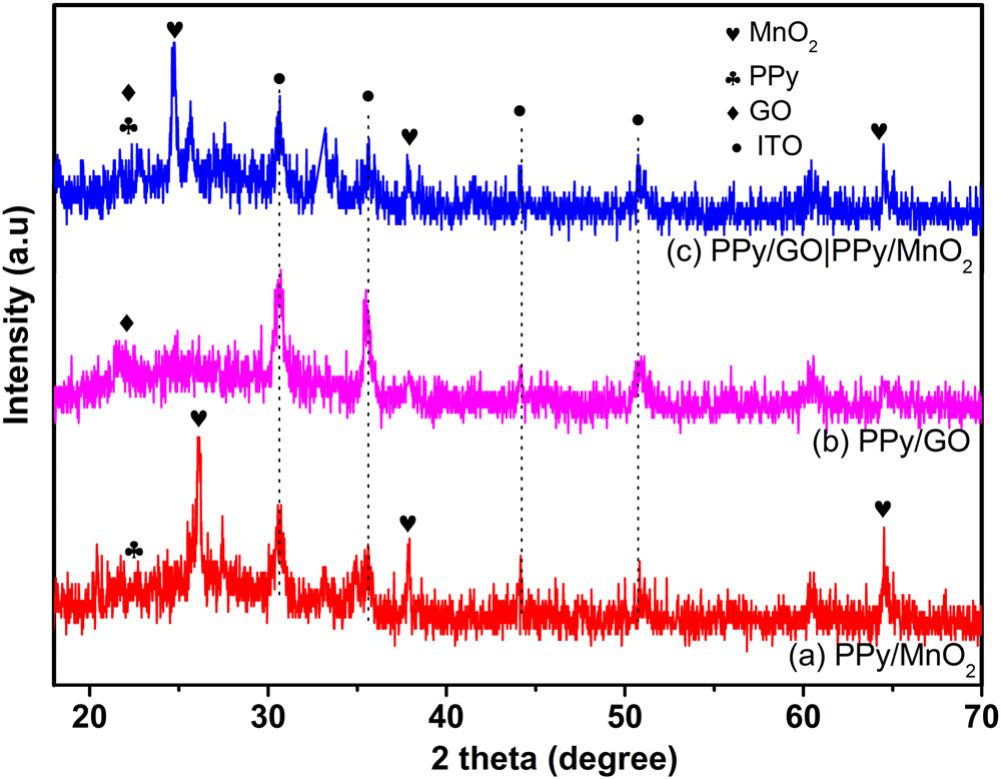
XRD patterns of the (**a**) PPy/MnO_2_, (**b**) PPy/GO and (**c**) LBL assembled composites.

In order to study the crystalline structure, XRD measurements were conducted on PPy/MnO_2_, PPy/GO and LBL assembled PPy/GO|PPy/MnO_2_, as shown in Fig. 4. The existence of MnO_2_ in LBL composite was further proved by comparing the LBL composite with MnO_2_ that obtained via electrodeposition under the same experimental condition. In the PPy/MnO_2_ spectrum (Fig. 4a), the peaks at 26.1° (002), 37.8° (100) and 64.6° (110) are ascribed to the characteristic of MnO_2_ (JCPDS No. 71-0071)^23^. These diffraction peaks correspond to tetragonal MnO_2_ phase. A weak and broad peak was observed in the spectra of PPy/MnO_2_ (Fig. 4a) and PPy/GO (Fig. 4) at 23–35° (002) indicating the diffraction peak of PPy and further suggesting that PPy is amorphous^24^. Moreover, the peaks for MnO_2_ are intense and distinguishable, showing that a highly crystalline phase of MnO_2_. Whereas, the X-ray analysis for the LBL assembled composite (Fig. 4c) shows all the characteristic peaks of PPy, GO and MnO_2_, confirming the LBL composite are made up of PPy/GO and PPy/MnO_2_.

### Field Emission Scanning Electron Microscope (FESEM)

**Figure 5:**
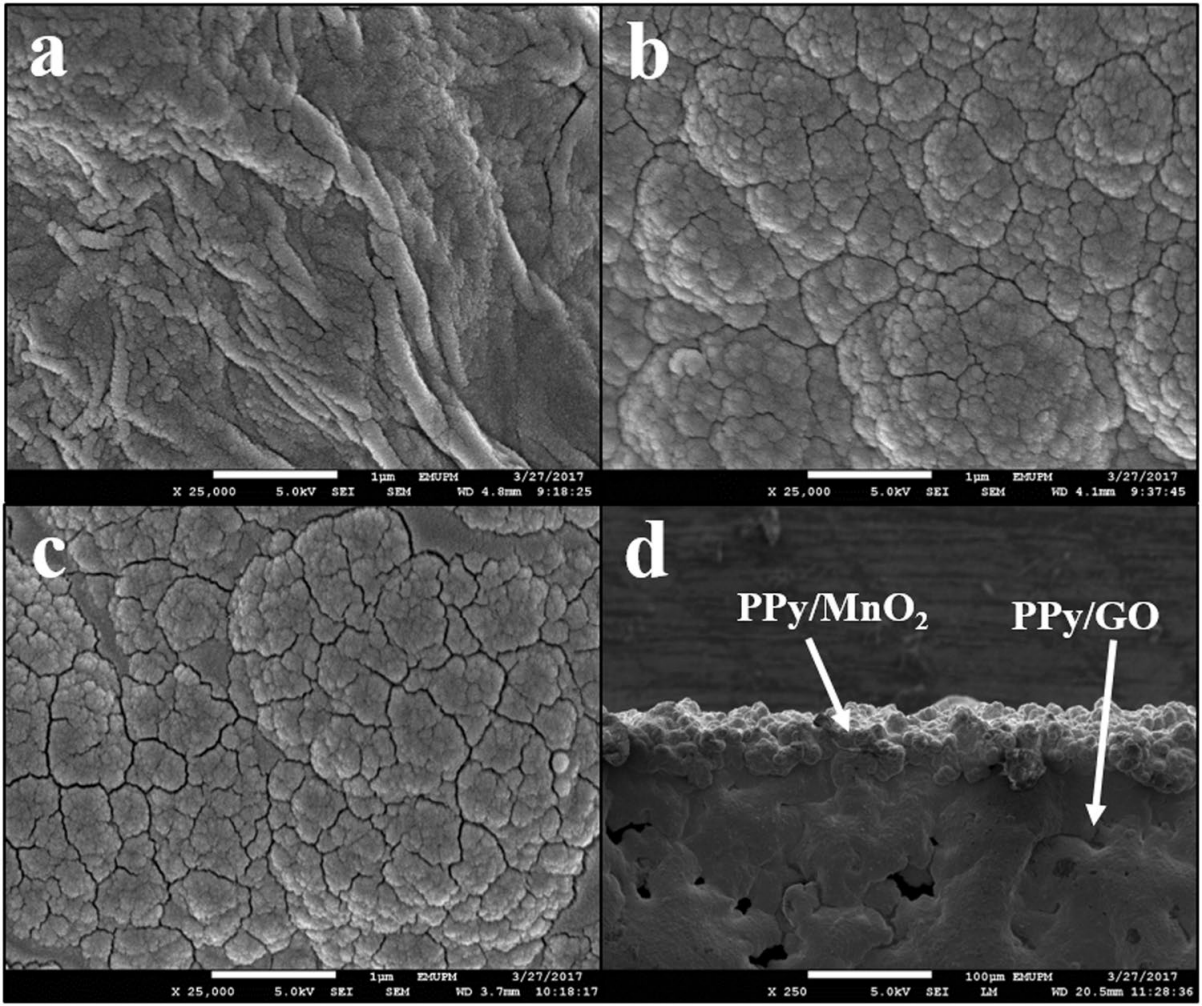
FESEM images of (**a**) PPy/GO composite, (**b**) PPy/MnO_2_ composite and (**c**) LBL assembled composite and (**d**) cross-sectional of LBL assembled composite.

FESEM was employed to study the morphologies of PPy/GO, PPy/MnO_2_ and LBL assembled composite. The image of PPy/GO displays rough and wrinkle surface (Fig. 5a) in which the PPy particles are uniformly grown together with GO sheets by hydrogen bonding and π − π interaction^16^. Whereas, Fig. 5b displays cauliflower like morphology indicating incorporation of MnO_2_ with PPy particles. This image shows a rough surface build by granular particles of composites which could provide a large surface area that enhance the performance of SC, as well as more charges can be stored. Utilizing LBL approach to electrodeposit PPy/MnO_2_ onto PPy/GO forming LBL assembled PPy/GO|PPy/MnO_2_ nanocomposite (Fig. 5c) which has similar morphology as PPy/MnO_2_ indicates PPy/MnO_2_ is successfully deposited on the surface on PPy/GO. This structure allows more ion diffusion and migration in the electrodes, implying high energy capacity^16^. Furthermore, the existence of MnO_2_ particles in the PPy matrix could increase the surface area and eventually is able to enhance the supercapacitive performance. The layered structure of LBL assembled composite can be confirmed with the cross-sectional view as shown in Fig. 5d. It is clearly seen that the film has two layers owing to PPy/GO and PPy/MnO_2_.

### Cyclic voltammetry

**Figure 6:**
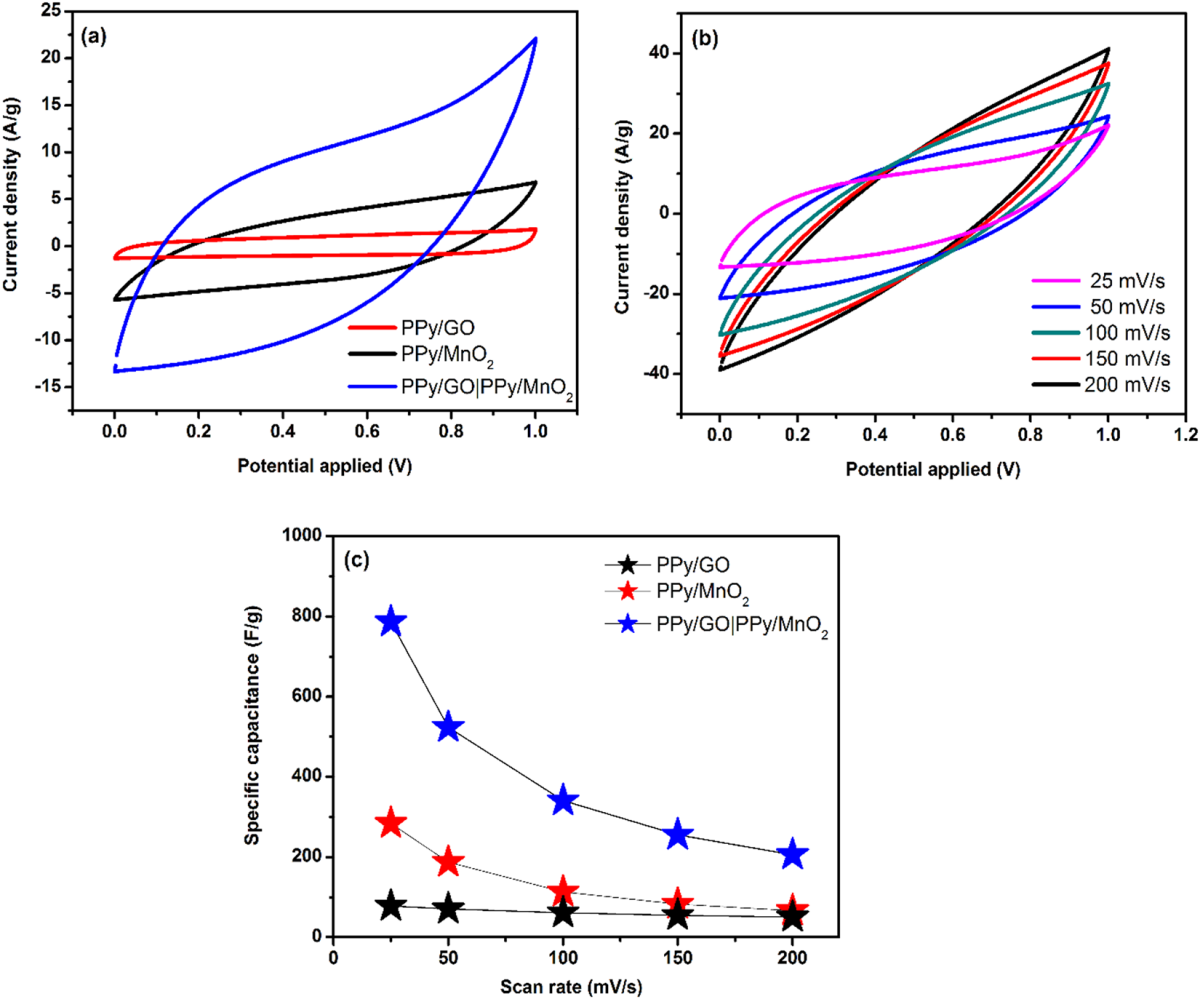
(**a**) CVs of PPy/GO, PPy/MnO_2_ and LBL assembled composite at a scan rate of 25 mV/s, (**b**) CVs and (**c**) C_sp_ of LBL assembled composite at scan rate ranges from 25 to 200 mV/s.

In order to investigate the electrochemical behavior of the as-prepared PPy/GO| PPy/MnO_2_ composite, PPy/GO and PPy/MnO_2_, cyclic voltammetric measurements were carried out using the two-electrode electrochemical system. Figure 6a shows the CV curves of LBL assembled composite, PPy/GO and PPy/MnO_2_ at a scan rate of 25 mV/s in a potential range of 0 to 1 V. The CVs of LBL assembled composite and PPy/MnO_2_ are quasi-rectangular, implying involvement of faradaic reaction, while the CV curves of PPy/GO is close to rectangular shape, indicating an ideal electrical double layer capacitor (EDLC) behavior^25^. It is found that *C*_sp_ of LBL assembled composite (786.6 F/g) is higher than single layer composites, PPy/MnO_2_ (284 F/g) and PPy/GO (78 F/g). The LBL assembled PPy/GO|PPy/MnO_2_ also has a higher *C*_sp_ compared to the ternary GO/PPy/MnO_2_ composite (207 F/g)^26^. This indicates that LBL assembled composite provides more active sites which enhance the electrochemical performance compared to the ternary composite. As shown in Fig. 6b, the CVs of LBL assembled composite at various scan rates (25 to 200 mV/s) exhibits quasi-rectangular shapes and the CV shape remain unchanged from the lowest scan rate (25 mV/s) to the highest scan rate (200 mV/s), implying ideal SC behaviour^27^. Moreover, the anodic and cathodic currents increase with the increasing of scan rate. However, the *C*_sp_ of LBL assembled bilayer composite decreases from 25 mV/s (786.6 F/g) to 200 mV/s (206 F/g) as shown in Fig. 6c due to limited ionic diffusion in the electrode material at high scan rate^28^.

### Galvanostatic charge-discharge (GCD)

**Figure 7:**
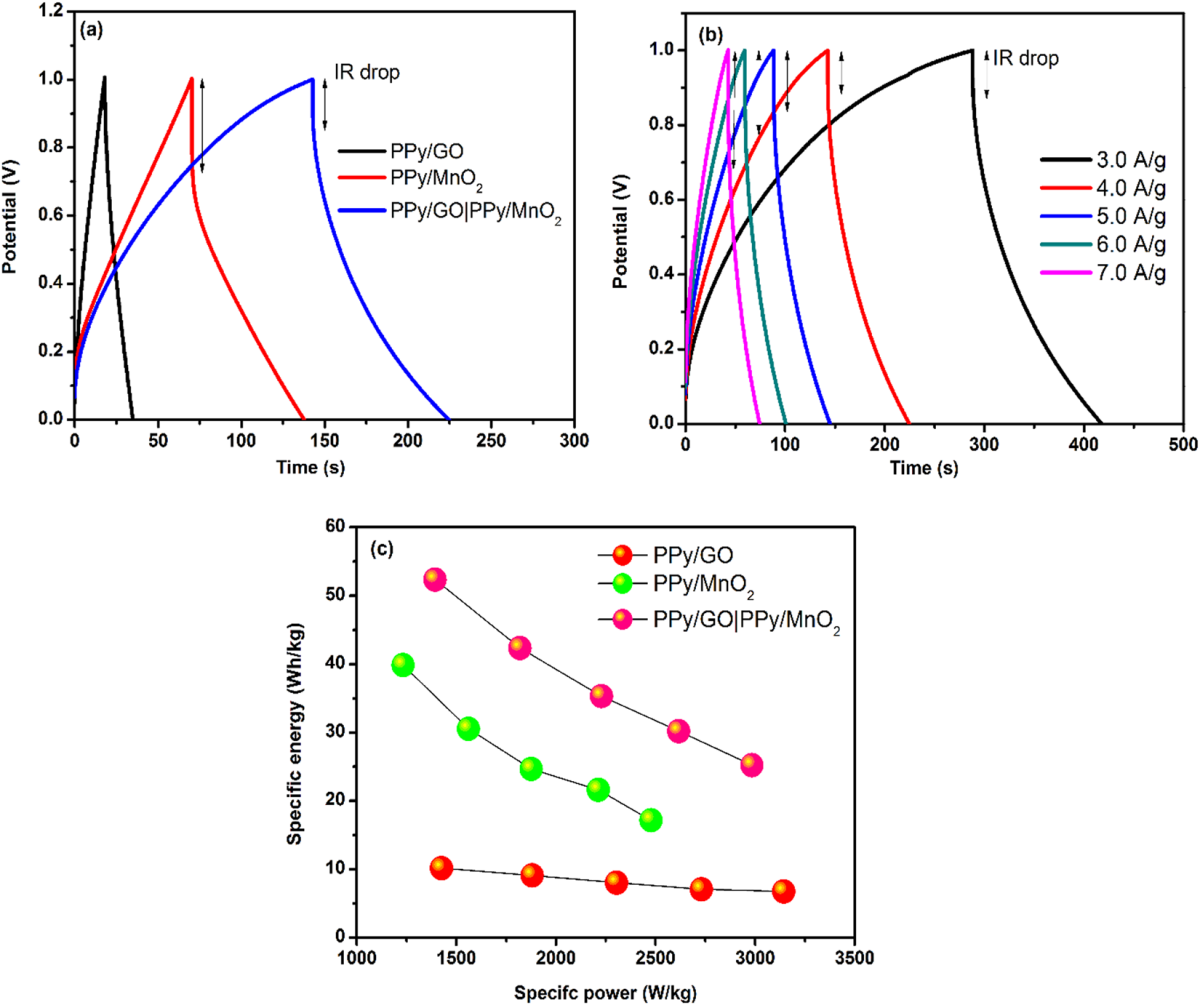
(**a**) GCD curves of LBL assembled composite, PPy/MnO_2_ and PPy/GO at current density of 4.0 A/g, (**b**) GCD curves of LBL assembled composite at current densities of 3.0 to 7.0 A/g and (**c**) Ragone plot of LBL assembled composite, PPy/MnO_2_ and PPy/GO composite electrodes.

Figure 7a shows the GCD curves of LBL assembled composite, PPy/GO and PPy/MnO_2_ at a current density of 4.0 A/g. Based on the GCD curves, PPy/MnO_2_ shows asymmetry triangular shape, whereas PPy/GO has symmetrical triangular shape. Meanwhile, the large distortion of the triangular shape of LBL assembled composite is mainly due to the domination of pseudocapacitive properties in the combination of both composites^29^. It is observed that LBL assembled composite has a longer discharging time compared to both single layer composites, indicating better capacitance behavior.

As shown in Fig. 7b, GCD curves of LBL assembled composite displays asymmetrical triangular shapes from the highest (7.0 A/g) to the lowest (3.0 A/g) current density which indicates the material has good charge-discharge reversibility^30^. However, the discharging time decreases with the increase of current density due to the incapability of the electrolyte ions to enter into the inner structure of the active material and only the outer active surface is utilized for ion diffusion at high current densities^16,31^. Figure 7c shows the Ragone plot (specific power vs. specific energy) of LBL assembled composite, PPy/MnO_2_ and PPy/GO. The specific energy is inversely proportional to specific power. The LBL assembled shows the highest specific energy (52.35 Wh/kg) at a specific power of 1392.90 W/kg compared to single layer composites, PPy/GO (10.12 W/kg) and PPy/MnO_2_ (39.83 W/kg) at specific power of 1232.74 Wh/kg and 1425.39 Wh/kg, respectively. This indicates that properties of LBL assembled composite give significant effect in the energy performance by combining active materials of PPy/GO and PPy/MnO_2_.

### Electrochemical Impedance spectroscopy

**Figure 8:**
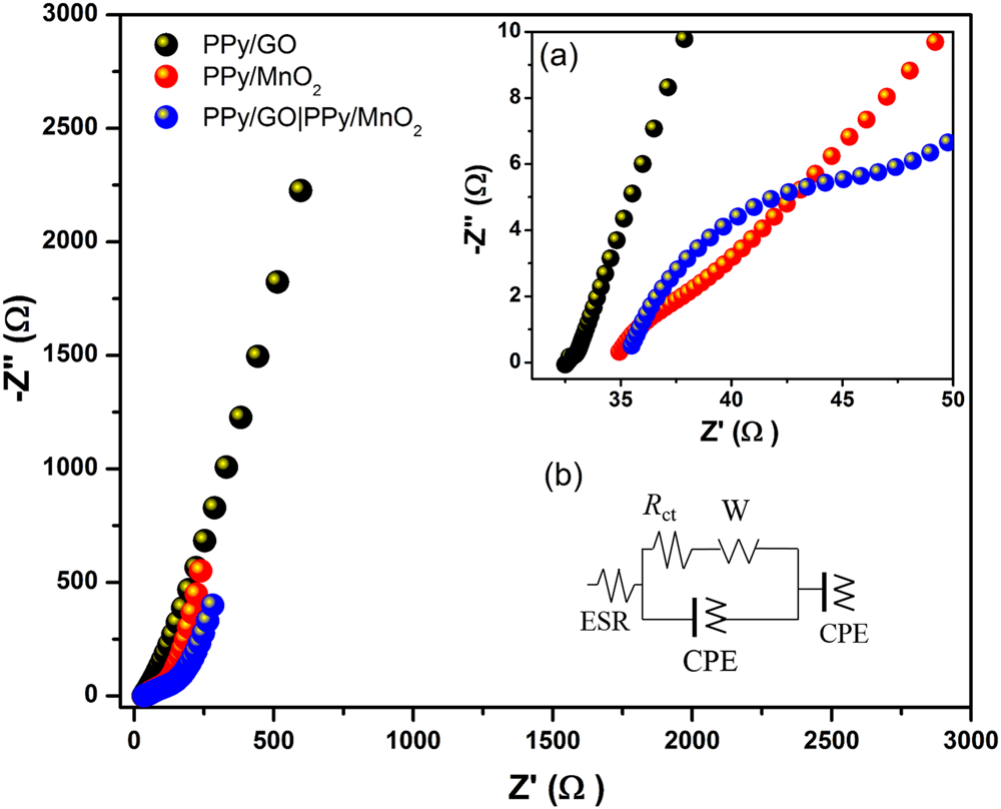
Nyquist plot of PPy/GO, PPy/MnO_2_ and LBL assembled composite with (**a**) the magnified view of the Nyquist plot at high frequency and (**b**) equivalent circuit.

The electrochemical performance of LBL assembled composite, PPy/GO and PPy/MnO_2_ was examined by electrochemical impedance spectroscopy **(**EIS) which were carried out over a frequency range from 100 kHz to 0.01 Hz at open circuit potential (Fig. 8). The Nyquist plot (imaginary component (−Z″) versus the real component (Z′)) shows the frequency response in the electrode material/electrolyte system^32^. All impedance spectra exhibited a vertical line approaching 90 degrees in the low frequencies region which corresponds to an ideal capacitor and fast ion diffusion in electrode materials^27^. Based on the magnified view of the Nyquist plot (Fig. 8a), a semicircle is also observed in the high-frequency range for all impedance spectra, indicating there is a hindrance in transferring charge at the interface^33^. Technically, the diameter of semicircle is associated with the charge transfer resistance (*R*_ct_) and the equivalent series resistance (ESR) is obtained at the intercept point of the real-axis at high-frequency region, which is related to the electrolyte resistance, the interfacial contact resistance between current collectors and active materials and the resistance of active materials^34^. Given in Fig. 8b, the equivalent circuit composes of ESR, *R*_ct_, constant phase element (CPE) for the irregular morphologies^35,36^, and the “classical” finite-length Warburg diffusion element (W) for the diffusion of electrolyte^37^ is used to fit the Nyquist plot. It is clearly observed that LBL assembled composite shows a higher *R*_ct_ (22.62 Ω) and ESR (35.13 Ω) compared with single layers. The high ESR value is an indication of poor contact between current collector and active materials, high intrinsic resistance of active materials and high ionic resistance of electrolytes^38^, whereas high *R*_ct_ value is due to the high resistance of the movement of ions at electrolyte/electrode interface^39^ as a result of the combined resistances of both single layers.

### Stability

**Figure 9:**
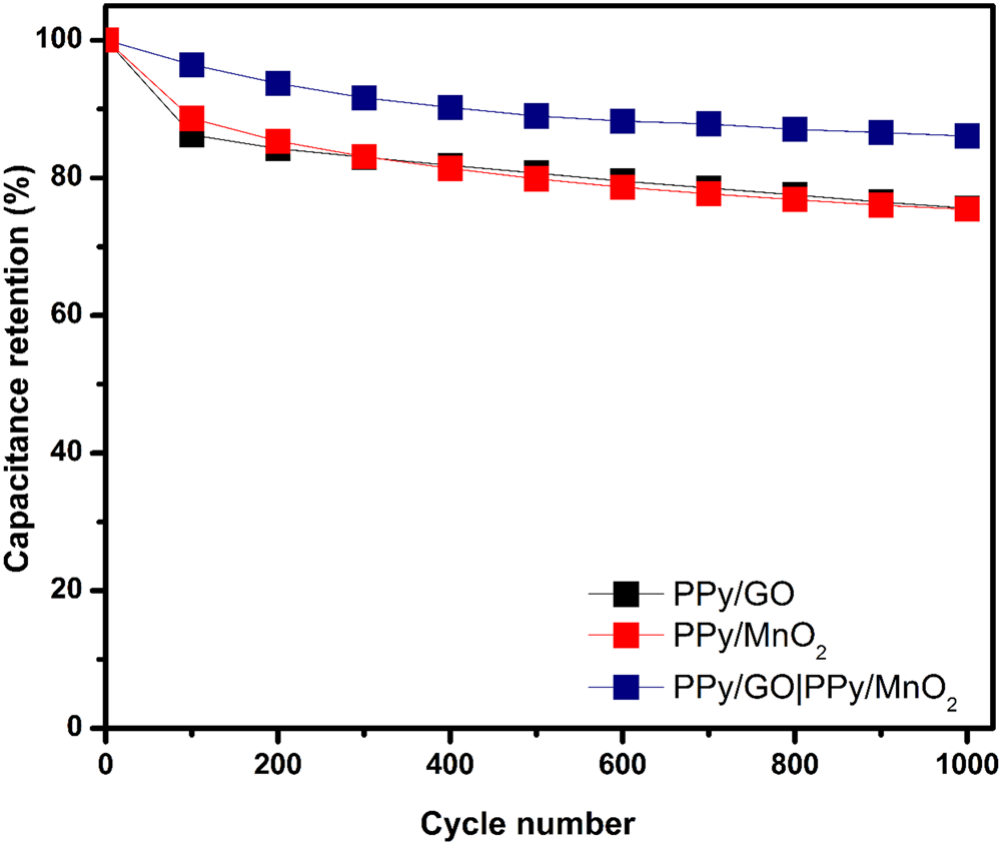
Specific capacitance retention of PPy/GO, PPy/MnO_2_ and LBL assembled composite after 1000 cycles at a scan rate of 100 mV/s.

The study on the electrochemical stability of the composites is another important parameter in evaluating the SC performance in real applications. After 1000 consecutive cycles (Fig. 9), the LBL assembled composite is able to retain exceptional cycling stability of 86.09% of its initial *C*_sp_ compared with both single layer composites, PPy/GO (75.58%) and PPy/MnO_2_ (75.44%). These results indicate that contribution of LBL assembled PPy/GO|PPy/MnO_2_ not only on promising *C*_sp_, specific energy and specific power but also improves the stability of the composite which significantly decreases the destruction of electroactive materials^40^ and significantly improves the stability during doping/dedoping process. The comparison with the previously published results (Table 1) indicates that our current work revealed outstanding performance and demonstrating that LBL assembled PPy/GO|PPy/MnO_2_ is a promising electrode in enhancing the performance of supercapacitor.

**Table 1:** Comparison of LBL assembled PPy/GO|PPy/MnO_2_ with previously reported literature.

Material	*C*_sp_	*E*	*P*	Stability	Electrolyte	Ref.
rGO/PPy	147.9 F/cm^3^ at 5 A/cm^3^	13.15 mWh/cm^3^	1300 mW/cm^3^	71.7% over 5000 cycles	PVA/H_2_SO_4_	^41^
MnO_2_/rGO	267 F/g at 0.2 A/g	17 Wh/kg	2520 W/kg	92.0% over 7000 cycles	PVA/H_3_PO_4_	^42^
PPy/MnO_2_ nanotube	403 F/g at 1 A/g	—	—	88.6% over 800 cycles	1 M Na_2_SO_4_	^43^
MnO_2_/PPy/rGO	404 F/g at 0.25 A/g	—	—	91.0% over 5000 cycles	1 M Na_2_SO_4_	^17^
rGO/MnO_2_/PPy	682 F/g at 5 mV/s	—	—	86.0% over 1000 cycles	1 M Na_2_SO_4_	^44^
PPy/GO|PPy/MnO_2_	786.6 F/g at 25 mV/s	52.3 Wh/kg	1392.9 W/kg	86.1% over 1000 cycles	1 M Na_2_SO_4_	This work

## Conclusion

LBL assembled PPy/GO|PPy/MnO_2_ was successfully prepared via a simple electrodeposition approach and used as electrode material for supercapacitor with a significant improvement of performance. The uniform thin film showed a high specific capacitance of 786.6 F/g at 25 mV/s, with the high specific energy of 53.3 Wh/kg at a specific power of 1392.9 W/kg, which are promising values for a high-performance supercapacitor. PPy/GO|PPy/MnO_2_ composite gained the advantage of LBL assembly, is apt to be an electrode for supercapacitor.
